# A Unique Case of Intraabdominal Polyorchidism: A Case Study

**DOI:** 10.1155/2016/2729614

**Published:** 2016-07-13

**Authors:** Javier Otero, Natalie Ben-Yakar, Biruk Alemayehu, Steven D. Kozusko, Frank Borao, Thomas S. Vates III

**Affiliations:** ^1^Department of Surgery, Monmouth Medical Center, Barnabas Health, Long Branch, NJ 07740, USA; ^2^Drexel University College of Medicine, Philadelphia, PA 19129, USA; ^3^St. George's University School of Medicine, St. George's, Grenada; ^4^Department of Urology, Monmouth Medical Center, Barnabas Health, Long Branch, NJ 07740, USA

## Abstract

*Background*. Polyorchidism, alternatively supernumerary testes (SNT), is a condition where an individual is born with more than two testicles. This congenital anomaly is quite rare and the literature has described various presentations.* Questions/Purposes*. To our knowledge, this presentation of polyorchidism has yet to be described in the literature. The goal of this case study is to add to the pediatric, general, and urologic surgery's body of knowledge of the subject matter.* Case Study*. A nine-month-old boy was admitted for an impalpable right testis and phimosis. At the time of surgical exploration, there appeared to be polyorchid testis on the right-hand side, with three masses that potentially appeared to be undescended testes.* Discussion*. Proponents of a conservative approach argue that infertility is common in patients with polyorchidism and, by preserving a potentially functional SNT, there may be improved spermatogenesis. When performing definitive surgical treatment, meticulous intra-abdominal and intrainguinal exploration must be undertaken. Orchiopexy should be performed to reduce the chances of torsion, malignancy, and infertility.* Conclusion*. Our case is important to the literature as it is the first known case of polyorchidism with 3 SNT on the right side, located intra-abdominally, and in a patient less than 1 year of age.

## 1. Introduction

*Background*. Polyorchidism, alternatively supernumerary testes (SNT), is a condition where an individual is born with more than two testicles [1]. A rare congenital anomaly which the literature has described in various presentations. The condition may present as unilateral or bilateral and ranges from partial to complete duplication. The literature reports vary but only up to two hundred instances of polyorchidism have been described [1, 2]. It often affects the left side and one study reports that only 25% of the cases are right-sided [3]. SNT is mostly scrotal (75%) with only a few reported cases of intra-abdominal polyorchidism (5%) [4].

This report presents the first known case of polyorchidism with three right-sided, intra-abdominal SNT in a patient less than one year of age.

*Presentation and Associated Anomalies*. Most commonly patients present with pain and swelling at a median age of 17 years [3]. The majority of patients with SNT have triorchidism with two testes in the scrotal sac [5]. However the differential diagnoses to SNT, which may be present simultaneously, or alternatively complicate the situation, include epididymal cyst and spermatocele [6].

The literature describes associated anomalies found concomitantly with SNT, including cryptorchidism, ectopic testis, hydrocele, indirect inguinal hernia, testicular torsion, and epididymitis [1, 3–8]. Found up to 30% of the time, indirect inguinal hernias frequently complicate the presentation of SNT, as they require repair [6, 7]. Similarly, testicular torsion is present in approximately 15% of cases and also always requires repair [2]. Only 16% of patients with SNT present without any symptoms [3, 5]. It is important to rule out neoplasm, as it has been shown to be associated in as many as 6% of cases [6–8]. Multiple types of neoplasms have been described, including seminomas, choriocarcinomas, and teratomas [3].

*Embryopathogenesis and Embryoetiology*. Understanding the embryology of testicular development elucidates the etiology of polyorchidism. In normal embryological development, the epididymis and vas deferens arise from the Wolffian duct [3]. In polyorchidism, a duplication or division of the genital ridge occurs. One theory for the development of SNT is that there is incomplete degeneration of a portion of the mesonephros and subsequent development of peritoneal bands [3]. These bands in turn cause transverse division of the genital ridge [5]. For an unknown reason, in polyorchidism the left genital ridge is more frequently affected than the right one [2].

## 2. Case Study

The patient is a 7.3 kg nine-month-old boy who was admitted by his parents for an impalpable right testis and phimosis. The boy had no prior medical or surgical history. His birth and first year of life were uncomplicated. All of his immunizations were up to date and he did not take any regular medication. He had no known allergies or family history. On physical examination, there was a palpable left testis but there was no palpable testis on the right.

Intraoperatively, an abnormal presentation of cryptorchidism was noted. Upon initial inspection, three masses appeared to be potentially undescended testes. The first was in the upper pelvis just posterior to the right internal inguinal ring. Two additional masses were noted superiorly along the right abdominal wall, adjacent to the liver (Figures 2, 3, and 4). All three masses contained a similar vascular pedicle which derived from the pelvic mass's vasculature. An atretic vas could be seen emanating from the two abdominal masses. There were multiple retroperitoneal adhesions, especially as the vascular pedicles coursed behind the colon. The smallest of the three masses was excised and sent for pathologic examination after ligating its vessels.

**Figure 1:**
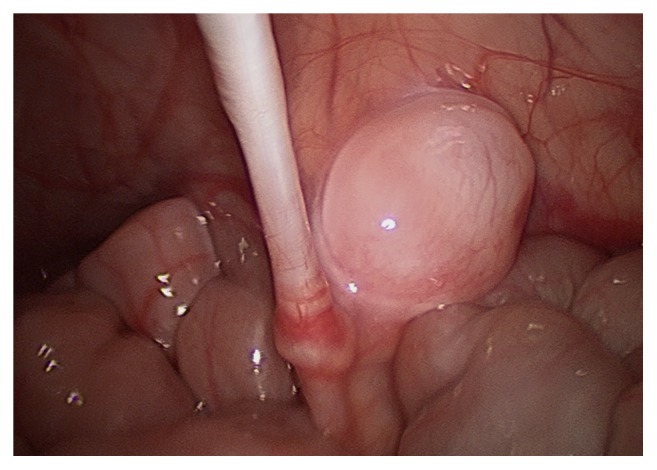
Testis with viable vas deferens noted just proximal to the right internal inguinal ring.

**Figure 2:**
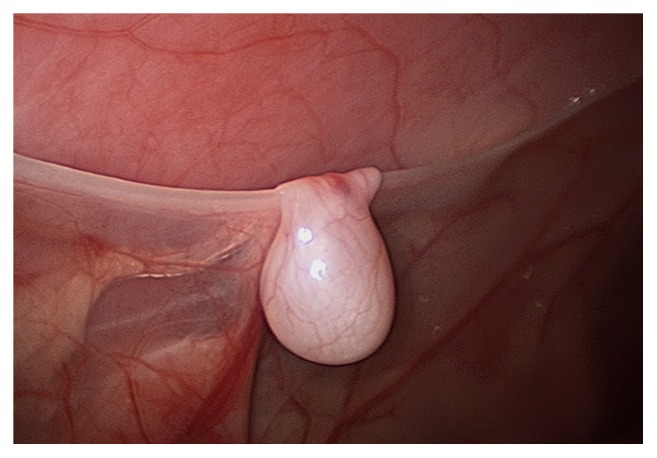
Testis located below peritoneal fold on the anterior abdominal wall with no visible associated vas deferens.

**Figure 3:**
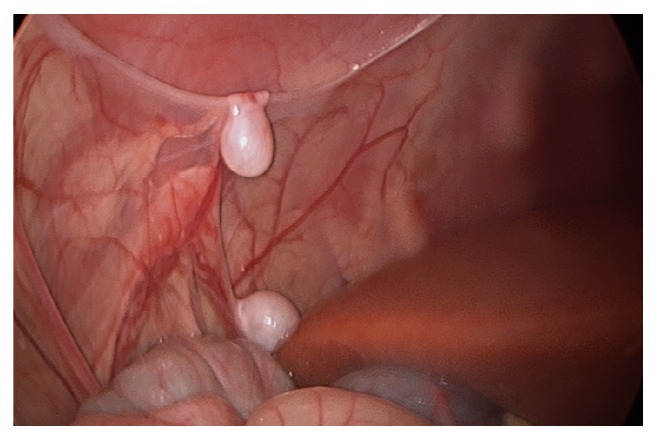
Testis in vicinity of liver (below), and testis on anterior abdominal wall.

**Figure 4:**
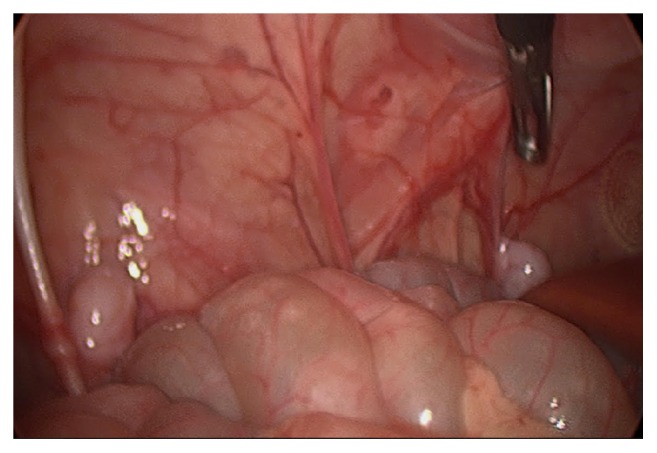
Most developed testis with viable vas at level of internal ring and relationship to testis near liver. Note, the third SNT has already been excised but would be located along the anterior abdominal wall within the vicinity of the bowel grasper.

Only one of the two remaining testicles appeared to have a viable vas deferens and was mobilized to the ipsilateral internal inguinal ring (Figure 1). The second, the higher one of the two testicles, was clipped and then excised for pathological examination. Further mobilization of the vas deferens down into the pelvis allowed for adequate mobilization of the last remaining right-sided cryptorchid testicle. It was determined that there was enough mobilization on this testicle to allow for an orchiopexy. An incision was made over the scrotum and a 5 mm trocar was inserted transscrotally through the inguinal region to allow for delivery of the testicle into the scrotum.

**Figure 5:**
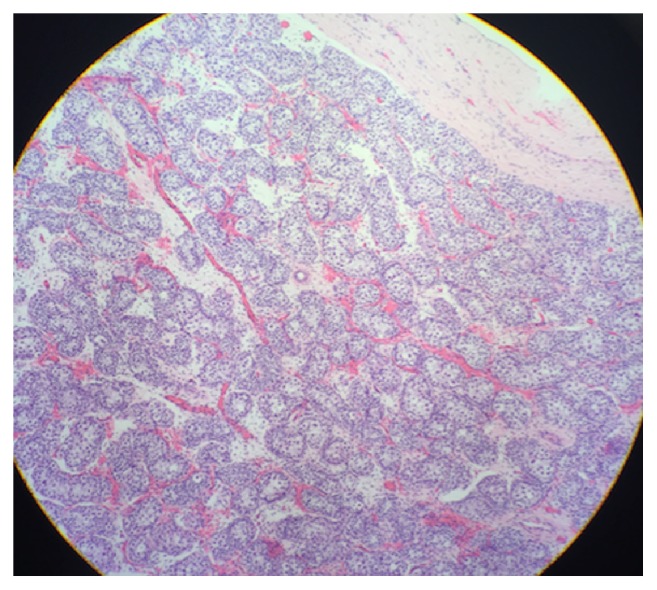
Histology confirmation of normally appearing testis.

Histologic evaluation of the specimens revealed that both were normally appearing testicles (Figure 5).

## 3. Discussion

### 3.1. Diagnostic Tools

When suspecting polyorchidism, one should begin the workup with an ultrasound (US) or magnetic resonance imaging (MRI) [1]. On Doppler ultrasonography, the SNT has similar echo texture and vascular flow as the normal testis [2]. On MRI, there is intermediate signal intensity on T1 and high signal intensity on T2 weighted images [2]. One of the difficulties in relying on ultrasound for diagnosis is that it may miss detecting polyorchidism and instead misdiagnose it as a cyst [3]. Another option for detection of SNT in an older patient is a CT scan, while other studies state that, with an equivocal US and MRI, one should proceed to surgery [5]. It is important to note, however, as was the case in this report, that the majority of cases are found intraoperatively in an asymptomatic patient [3].

### 3.2. Surgical Techniques and Descriptions

When discovering SNT, there are arguments for and against surgery. Proponents of a conservative approach argue that infertility is common in patients with polyorchidism and, by preserving a potentially functional SNT, there may be improved spermatogenesis [7]. This, however, must be weighed against the risk of malignancy. When choosing the conservative route, MRI surveillance is essential [7]. This conservative approach has gained support given the improvements in advanced imaging.

Proponents of surgical removal argue that the risks of malignancy as well as torsion are reasons to remove SNT. One study argues that the treatment for SNT should be laparoscopic intra-abdominal exploration followed by inguinal exploration on the affected side [9]. Laparoscopy helps to identify the SNT, epididymis, vas deferens, and vessels.

### 3.3. Management

Definitive treatment depends on many factors. If there are suspicious findings on advanced imaging, it is prudent to remove the SNT [1, 2]. Inguinal and intra-abdominal SNT has a higher risk of tumor formation compared to intrascrotal one [1]. In cases where the SNT is intrascrotal with no imaging abnormalities, the patient can be managed conservatively [1]. Nonetheless, patients managed nonoperatively may develop scrotal pain, necessitating eventual operative removal [2].

Another way to approach treatment of SNT is based on the vas deferens. Patients with an intact vas deferens may have fertility potential while SNT without a vas has no potential to contribute to spermatogenesis [3]. When performing definitive surgical treatment, meticulous intra-abdominal and intrainguinal exploration must be undertaken. Orchiopexy should be performed to reduce the chances of torsion, malignancy, and infertility [4].

In this case, the patient had multiple right-sided intra-abdominal SNT, which poses a higher risk for future malignancy. Furthermore, we were able to mobilize the most developed of the testes with an intact vas deferens into the scrotal sac. As is most frequently the case, the patient was discovered to have the condition intraoperatively.

## 4. Conclusion

Polyorchidism or SNT is a rare congenital condition with many associated anomalies. It is important to always rule out neoplasm in patients identified with SNT. This condition has only been described up to 200 times in the literature. To our knowledge, no other case of polyorchidism with a total of four testes has been described. Furthermore, our patient had a right-sided presentation with three testes in the abdominal cavity. We hope that this case study illustrates the variability of polyorchidism and contributes to the limited body of knowledge about its presentation in pediatric patients.
